# Association Between Natural Lithium Exposure and Suicide Rate: An Ecological and Biomonitoring Study in Portugal

**DOI:** 10.3390/nu17071283

**Published:** 2025-04-07

**Authors:** Carolina Gonçalves, Rui Azevedo, Cristina Couto, Mary Duro, Agostinho Santos, Laura Cainé, Agostinho Almeida

**Affiliations:** 1 Faculty of Medicine, University of Porto, 4200-319 Porto, Portugal; carolina.isgoncalves4@gmail.com (C.G.); agostinho.c.santos@inmlcf.mj.pt (A.S.); laura.m.caine@inmlcf.mj.pt (L.C.); 2 LAQV/REQUIMTE, Department of Chemical Sciences, Faculty of Pharmacy, University of Porto, 4050-313 Porto, Portugal; ruiazevedo43@gmail.com (R.A.); cristina.couto@iucs.cespu.pt (C.C.); mduro@ufp.edu.pt (M.D.); 3 Associate Laboratory i4HB, Institute for Health and Bioeconomy, University Institute of Health Sciences—CESPU, 4585-116 Gandra, Portugal; 4 UCIBIO-Applied Molecular Biosciences Unit, Forensics and Biomedical Sciences Research Laboratory, University Institute of Health Sciences (1H-TOXRUN, IUCS-CESPU), 4585-116 Gandra, Portugal; 5 Nordestelab Clinical Analysis Laboratory Dra. Matilde Sampaio, 5300-252 Mogadouro, Portugal; 6 Higher School of Health, Fernando Pessoa Foundation, 4200-253 Porto, Portugal; 7 National Institute of Legal Medicine and Forensic Sciences, North Branch, 4050-167 Porto, Portugal; 8 School of Health Sciences, Minho University, 4710-057 Braga, Portugal

**Keywords:** lithium, natural exposure, suicide rate, urine, biomonitoring

## Abstract

**Background/Objectives**: Higher lithium (Li) levels in drinking water have been linked to lower suicide rates in the general population in several ecological studies, though this relationship is not always consistent. The main limitation of such studies is the assumption that Li content in drinking water is directly correlated with total Li intake, which may not always be the case for several reasons. **Methods**: In this context, we conducted a biomonitoring study to compare urinary Li levels—assumed as a reliable indicator of total Li intake—among individuals from three regions with different suicide relative risks (RRs): Porto Metropolitan Area (PMA; low RR), Central region (CT; intermediate RR), and Trás-os-Montes region (TM; high RR). Each participant provided a urine sample (first morning) and two water samples (drinking water and environmental water). Li concentrations were determined using ICP-MS. **Results**: A total of 311 individuals participated in this study. The median (P25–P75) urinary Li concentration was 21.9 (15.1–46.0) in PMA, 19.0 (12.6–30.4) in CT, and 24.2 (14.6–38.7) µg/L in TM, with no statistically significant differences between regions (Kruskal–Wallis test with Bonferroni correction). Urinary Li was weakly correlated with Li in drinking water (ρ = 0.174; *p* = 0.002) but not with Li in environmental water (ρ = −0.036; *p* = 0.694). **Conclusions**: These findings do not support a protective role of natural Li exposure in suicide risk at the low levels found in drinking (P75 = 3.75 µg/L) and environmental (P75 = 6.87 µg/L) water. More robust and comprehensive biomonitoring studies are needed to clarify the potential impact of natural Li exposure on suicide rates.

## 1. Introduction

Suicide is a major public health concern, with an estimated 700,000 deaths worldwide each year. And for every completed suicide, more than 20 attempts are estimated to occur [1]. According to the latest World Health Organization data, Portugal’s suicide rate in 2019 was 11.5 per 100,000 inhabitants [2]. The propensity for suicide is a complex phenomenon, influenced by multiple external factors beyond individual conditions [1].

Lithium (Li) is a relatively rare alkali metal discovered in 1817 [3]. Its efficacy as a therapeutic agent became evident in the 1950s for treating mania, and since then, its oral administration (usually as lithium carbonate) has been widely used as a mood stabilizer, particularly in the treatment of bipolar disorder [4,5]. For this purpose, maintenance doses generally range from 900 mg to 1800 mg lithium carbonate per day, which corresponds to approximately 170–340 mg elemental Li [6]. Therapeutic blood levels are 0.8–1.2 mmol/L (approx. 4.2–8.3 mg/L) for maintenance treatment and refer to the Li concentration regardless of the formulation used [7]. Lithium appears to exert its clinical effects by modulating excitatory and inhibitory neurotransmission and by targeting intracellular signalling pathways. However, the exact neural and molecular mechanisms of action of Li are still unknown [8].

In nature, due to its high reactivity, Li is always found as part of various minerals (in rocks) and is unevenly distributed [3,5]. From these minerals, Li is gradually released into water and soil and later absorbed by plants, thus entering the food chain [5]. Daily intake from natural sources has been estimated between 7 and 29 µg/day [9].

In 1990, using data on Li levels in drinking water for the period 1978–1987 in Texas (USA), Schrauzer and Shrestha observed significantly higher rates of suicide, homicide, and rape in counties with low Li levels (<12 µg/L) compared with counties with high levels (70–170 μg/L) [10]. Since then, several systematic reviews and meta-analyses of ecological studies have reported an inverse correlation between Li levels in drinking water and suicide mortality rates in the general population [11,12,13,14,15], i.e., regions with higher Li levels in drinking water tend to have lower suicide rates [16,17,18]. However, not all studies have observed this association [19,20,21], and it appears that this putative protective effect of Li is not observed for Li concentrations in drinking water below 30 µg/L [19,21].

A key limitation of ecological studies is the assumption that Li levels in drinking water correlate with actual Li intake by the general population, overlooking the importance of other factors [14,20,22]. The rationale is that if drinking water is rich in Li, so is the environment and food produced in the region and, therefore, the total Li intake by the population. A second key limitation is the assumption that populations primarily consume locally sourced drinking water and food. However, modern dietary habits often involve the consumption of drinking water and food sourced from outside the area of residence, purchased in stores of global supermarket chains, which greatly weakens the correlation between drinking water Li levels and actual population Li intake.

To address the first limitation, it is essential to measure Li levels not in drinking water but in environmental waters (both surface and groundwater), as they allow for characterizing the local geochemical environment in terms of Li richness, as well as indirectly characterizing the Li richness of locally produced food. To overcome the second limitation (external origin of food), it becomes mandatory to carry out biomonitoring studies (direct analysis of human biological samples), as suggested by Prazeres et al. (2019) [22]. Since Li is excreted mainly in urine, determining urinary Li levels appears to be the best strategy to assess the magnitude of the natural exposure and, therefore, the total daily intake of Li by the general population.

Using statistical data on suicide mortality for the period 1980–2015, Loureiro et al. (2018) [23] created a “spatiotemporal clusters” map for mainland Portugal, identifying regions with very different relative risks (RRs) for suicide (Figure S1). Namely, the Trás-os-Montes region (cluster E in the original study) exhibited a high relative risk (RR = 1.67), the third highest in the country; a cluster (H) in the central region had an intermediate RR (0.58), and a cluster (F) roughly corresponding to the Porto Metropolitan Area was the region with the lowest suicide RR (0.28).

In this context, the present study aimed to investigate whether an inverse relationship between suicide rate and Li intake is observed in Portugal. This study was conducted with individuals from the three regions referred to above, given the regional differences in suicide RR. In addition to drinking water, environmental water samples were also analysed to characterize the local geochemical environment in terms of Li levels. Furthermore, in what, to our knowledge, appears to be the first study of its kind, urine samples from individuals residing in the three regions were analysed to assess total Li exposure.

## 2. Materials and Methods

### 2.1. Sample Collection

This study was conducted in three regions of mainland Portugal with distinct relative risks (RRs) of suicide: Porto Metropolitan Area (PMA; low RR), Central region (CT; intermediate RR), and Trás-os-Montes region (TM; high RR). Data on suicide RR were taken from the study by Loureiro et al. (2018), based on statistical records available for the period between 1980 and 2015 [23] (Figure S1).

Participants were recruited between October 2022 and May 2023 through convenience sampling, starting within the researchers’ personal networks and expanding through snowball recruitment, where the initial participants invited others from their own social circles.

Each participant received a standard plastic container for urine collection (first morning urine) and two 15 mL plastic tubes for water collection. The participants were instructed to collect a sample of the water usually used for drinking and cooking (“drinking water”) in tube 1 and a sample of “environmental water” from a regional source (e.g., springs, fountains, rivers, lakes, wells, boreholes, etc.) in tube 2. The participants also completed a brief questionnaire providing information on sex, age, and zip code (to confirm residence in the study regions) as well as on the source of the drinking water sample (public water supply network, well, borehole, mine, etc.) and the environmental water sample (well, borehole, mine, river, lake, natural spring, etc.). This study was explained to all participants both orally and through an informational leaflet. All participants signed an Informed Consent Form. In addition to residing in the study regions, the inclusion criteria required the participants to be 18 years or older and apparently healthy (i.e., individuals with a known, medically diagnosed condition were excluded). This study was approved by the Ethics Committee of the Faculty of Pharmacy, University of Porto (Report n. 04-02-2022). Except during short transport periods, the samples were maintained at temperatures between 2 °C and 8 °C.

### 2.2. Sample Analysis

All solutions were prepared with ultrapure water (≥18.2 MΩ.cm at 25 °C) obtained from a Sartorius Arium^®^ Pro water purification system (Gottingen, Germany). Nitric acid (HNO_3_; 67–69% *w*/*w*, Primar Plus™, for Trace Metal Analysis) was obtained from Fisher Scientific (Loughborough, UK), Triton X-100 was obtained from Sigma-Aldrich (St. Louis, MO, USA), and absolute anhydrous ethanol was obtained from Carlo Erba Reagents (Val de Reuil, France).

All laboratory materials (bottles, tubes, volumetric flasks, etc.) were of polypropylene or high-density polyethylene (HDPE) and were thoroughly decontaminated by immersion in a 10% *v*/*v* HNO_3_ solution for at least 24 h, followed by extensive rinsing with ultrapure water and air-drying under dust-free conditions.

Trace element determinations were performed using an inductively coupled plasma mass spectrometer (ICP-MS) from Thermo Fisher Scientific (Waltham, MA, USA), model iCAP™ Q. The instrument was equipped with a TQ+ quartz concentric nebulizer (Meinhard^®^, Golden, CO, USA), a high-purity quartz cyclonic spray chamber, and a demountable quartz torch with a 2.5 mm internal diameter quartz injector. The interface consisted of two nickel cones (sampler and skimmer). High-purity argon (99.9997%) supplied by Gasin (Matosinhos, Portugal) was used as the nebulizer, auxiliary, and cool gas.

Before each analytical run, the instrument was tuned for maximum sensitivity and signal stability, minimizing oxides and doubly charged ions. The main operating parameters of the ICP-MS instrument were as follows: nebulizer gas flow rate, 1.08 L/min; auxiliary gas flow rate, 0.79 L/min; plasma gas flow rate, 13.9 L/min; radiofrequency generator power, 1550 W; and dwell time, 20 ms.

The urine samples were analysed following a procedure published by the U.S. Centers for Disease Control and Prevention (CDC) [24]. The samples were diluted 1:10 with a diluent solution containing 2% *v*/*v* HNO_3_, 500 µg/L Au (added from Gold Standard for ICP, 1000 mg/L, Fluka, Steinhein, Germany), 1.5% *v*/*v* ethanol, and 10 µg/L ^6^Li (added from Internal Standard Mix 1-SCP-IS7, 10 mg/L, PlasmaCAL, SCP Science, Baie D’Urfé, QC, Canada) as internal standard (IS). The water samples were analysed in a similar manner, except that the diluent solution did not contain ethanol.

For Li analysis, an eight-point calibration curve (0, 1.0, 5.0, 10, 25, 50, 100, and 200 µg/L) was prepared using standard solutions obtained by appropriately diluting a commercial multi-element solution (ICP multi-element standard solution XVI, 100 mg/L, Certipur^®^, Supelco, Steinhein, Germany). These calibration solutions were then diluted 1:10 with the same diluent used for the samples. For urine analysis, the calibration standards were matrix-matched with pooled urine samples, as recommended in [24]. Urine and water samples with Li concentrations outside the calibration range were further diluted. The elemental isotope ^7^Li was measured for analytical determination, while the isotope ^6^Li was monitored as the IS. Since samples naturally contain a mixture of ^6^Li and ^7^Li, the signal intensity of ^6^Li was corrected using the equation −0.072397 × ^7^Li.

A washing solution containing 5% *v*/*v* HNO_3_, 1.5% *v*/*v* ethanol, 0.002% *v*/*v* Triton X-100, and 500 µg/L Au was pumped through the sample introduction system between the urine samples to prevent carry-over effects. In the analysis of the water samples, a similar washing solution was used, but without ethanol.

After complete homogenization using a vortex mixer, diluted samples and calibration standards were introduced into the ICP-MS instrument using a CETAC ASX-520 autosampler (Teledyne CETAC Technologies, Omaha, NE, USA). For analytical quality control (QC), the certified reference material EnviroMAT Drinking Water EP-H (SCP Science) and the QC samples Seronorm™ Trace Elements Urine L-1 and L-2 (obtained from SERO AS, Billingstad, Norway) were periodically analysed (at the beginning, middle, and end of the analytical series). The quality control results are shown in Table S1. The limit of detection (LD) was estimated as 3 times the standard deviation of the blank.

### 2.3. Statistical Analysis

Statistical analysis was performed using SPSS (Statistical Package for the Social Sciences) version 29.0. The normality of continuous numeric variables was assessed using the Kolmogorov–Smirnov test. For non-normally distributed variables, comparisons between independent groups were performed by the non-parametric Mann–Whitney or Kruskal–Wallis tests (comparison between two or more than two groups, respectively), while for normally distributed variables, the Student’s *t*-test or ANOVA were used. Spearman’s correlation coefficient was used to assess the existence of an association between urinary Li levels and Li levels in drinking water or environmental waters. The Chi-squared test for independence was used to assess differences between groups in nominal variables. A significance level of 0.05 was adopted for all tests.

## 3. Results

The LD was 0.515 for Li in urine and 0.272 µg/L for Li in water. No urine samples had a concentration below the LD. Ten (3%) drinking water samples and two (1%) environmental water samples were below the LD.

A total of 311 individuals participated in this study: 91 (29%) resided in the PMA region (low RR), 130 (42%) in the CT region (intermediate RR), and 90 (29%) in the TM region (high RR) (Table 1). All participants (n = 311) provided a urine sample, 305 (98%) provided a drinking water sample, and 298 (96%) provided an environmental water sample.

Almost all of the participants (n = 292; 94%) reported their sex. Overall, the majority (n = 208; 67%) were women. This higher percentage of female participants was observed in all regions. There were statistically significant differences (*p* = 0.019) between the sex distribution of the three regions (Table 1). The mean age (standard deviation, SD) of the participants was 47 (17) years, ranging from 18 to 95 years. There were statistically significant differences between the age distribution of the three regions (*p* = 0.016).

**Figure 1:**
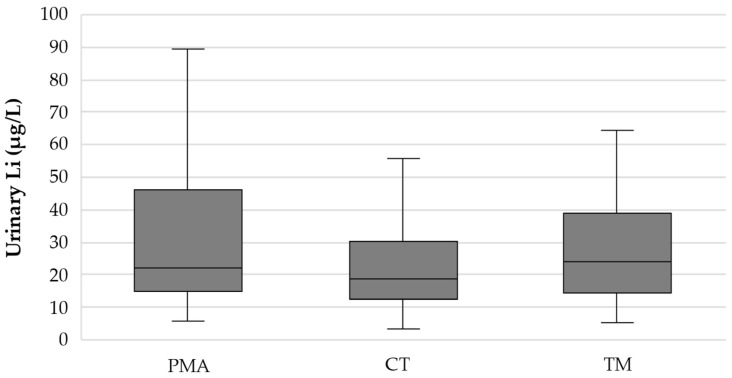
Box plot showing the distribution of the urinary Li concentration (µg/L) in the three study regions (PMA—Porto Metropolitan Area; low RR); CT—Central region; intermediate RR); TM—Trás-os-Montes region; high RR).

The median (P25–P75) urinary Li concentration in the PMA, CT, and TM regions was 21.9 (15.1–46.0), 19.0 (12.6–30.4), and 24.2 (14.6–38.7) µg/L, respectively (Figure 1). After applying the Bonferroni correction for multiple comparisons, no statistically significant differences were found between regions (PMA-CT: *p* = 0.119; PMA-TM: *p* = 1.00; CT-TM: *p* = 0.117).

A total of 305 participants provided a sample of drinking water (water used for drinking and cooking), and 300 (98%) also reported the water source. Of these, 244 participants (81%) provided samples from the public water supply network, while 18 participants (6%) provided samples from wells, boreholes, or other similar groundwater sources. Thirty-eight participants (13%) reported other water sources (Table 2). There were statistically significant differences (*p* < 0.001) between the three regions regarding their drinking water sources. No statistically significant differences (*p* = 0.445) were observed in the median (P25–P75) urinary Li concentration between the participants using drinking water from the public supply network [21.2 (14.0–36.3) µg/L], from wells, boreholes, or another groundwater source [16.4 (14.3–26.6) µg/L], or from other non-specified sources [21.1 (16.9–30.9) µg/L].

A total of 298 participants provided an environmental water sample, and 278 (93%) also reported the source of the sample. Most samples (n = 135; 49%) were from public fountains, 67 (24%) were from wells, boreholes, or other similar underground water sources, and 29 (10%) were from rivers, lakes, or other surface water sources. The remaining 47 (17%) samples were from non-specified sources (Table 2). Similar to the drinking water, there were statistically significant differences (*p* < 0.001) between the three regions regarding the sources of environmental water. No statistically significant differences (*p* = 0.957) were observed in the median (P25–P75) urinary Li concentration between the participants who provided the different types of environmental water samples: 22.7 (14.3–33.8) µg/L for public fountains, 20.1 (12.9–36.6) µg/L for wells, boreholes, or other similar underground water sources, 18.3 (14.0–39.1) µg/L for rivers, lakes, or other surface water sources, and 22.7 (15.5–33.7) µg/L for other non-specified sources.

**Figure 2:**
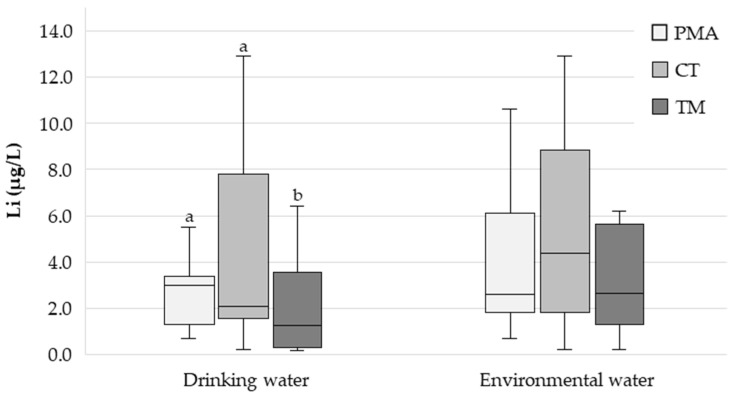
Box plot showing the distribution of the drinking water and environmental water Li concentration (µg/L) in the three study regions (PMA—Porto Metropolitan Area; low RR); CT—Central region; intermediate RR); TM—Trás-os-Montes region; high RR). Different letters indicate statistically significant differences (*p* < 0.05).

The median (P25–P75) Li concentration in the drinking water from the PMA, CT, and TM regions was 3.10 (1.38–3.27), 1.91 (0.925–6.43) and 1.24 (0.407–3.46) µg/L, respectively (Figure 2). Statistically significant differences were found between the three regions (*p* < 0.001). Pairwise comparisons revealed significant differences between the PMA and TM regions (*p* < 0.001) and between the CT and TM regions (*p* = 0.006) but not between the PMA and CT regions (*p* = 0.604). The Li concentration in the drinking water from the PMA region exhibited less heterogeneity (range: 0.658–29.6 µg/L) compared to the CT (<0.272–35.2 µg/L) and TM (<0.272–45.8 µg/L) regions.

The median (P25–P75) Li concentration in the environmental waters from the PMA, CT, and TM regions was 2.81 (1.83–6.10), 4.48 (1.82–8.61), and 2.77 (1.35–5.64) µg/L, respectively (Figure 2). The Li concentration in the environmental waters from the CT region exhibited greater heterogeneity (range: <0.272–666 µg/L) compared to the PMA (<0.272–111 µg/L) and TM (<0.272–101 µg/L) regions.

The urinary Li concentration showed a weak positive correlation with the Li concentration in the drinking water (Spearman’s rank correlation coefficient, ρ = 0.174; *p* = 0.002) but no correlation with the Li concentration in the environmental waters (ρ = −0.036; *p* = 0.694). The correlation between the Li concentrations in the urine and drinking water was strengthened after excluding the participants who provided drinking water samples with Li concentrations below 10 µg/L (ρ = 0.451; *p* = 0.046; n = 20).

No statistically significant differences were found between the urinary Li concentrations of men and women (*p* = 0.575). The median (P25–P75) urinary Li concentration was 20.9 (14.5–34.8) µg/L for men and 20.6 (14.1–36.1) µg/L for women.

## 4. Discussion

Several systematic reviews and meta-analyses of ecological studies have demonstrated a clear negative association between Li concentration in drinking water and suicide rates in the general population [11,12,13,14,15]. In other studies, however, this association was not observed [19,20,21].

The main limitation of ecological studies is that they overlook contributions from other sources and assume that Li levels in drinking water directly correlate with the actual population’s total Li intake, which may not be the case in modern urban environments [14,20,22]. On the one hand, Li levels in drinking water may not reflect the geochemical characteristics of a region, including Li levels in soil and water (both surface and groundwater), and, ultimately, in locally produced food. This is because, unlike in the past, when drinking water was obtained from local sources (fountains, wells, mines), it is now typically supplied through public water systems, often sourced from regions relatively far from residential areas. Additionally, most of the food consumed today is purchased in supermarkets and mostly also originates from outside the area of residence. As a result, the population’s dietary intake of Li may have no relationship at all with the Li richness of the local environment.

Thus, clarifying the possible relationship between natural exposure to Li and suicide rates in the general population requires a more comprehensive approach, including the assessment of actual intake, which entails biomonitoring studies, such as the determination of Li in urine.

In the present study, 311 urine samples from participants residing in three regions with quite different suicide RRs were analysed. Lithium is rapidly and extensively excreted through urine [25], making urine the most suitable biological sample for assessing daily Li exposure (intake). The median (P25–P75) urinary Li concentrations in the PMA (low RR), CT (intermediate RR), and TM (high RR) regions were 21.9 (15.1–46.0), 19.0 (12.6–30.4), and 24.2 (14.6–38.7) µg/L, respectively (Figure 1). Pairwise comparisons with Bonferroni correction for multiple tests proved the absence of statistically significant differences between regions (PMA-CT: *p* = 0.119; PMA-TM: *p* = 1.00; CT-TM: *p* = 0.117) and, therefore, no correlation between urinary Li levels and suicide RR. To our knowledge, no other study has compared urinary Li concentration across regions with different suicide RRs.

A total of 305 drinking water samples were analysed. The median (P25–P75) Li concentration was 3.10 (1.38–3.27) in the PMA region, 1.91 (0.925–6.43) in the CT region, and 1.24 (0.407–3.46) µg/L in the TM region (Figure 2). Interestingly, the difference was statistically significant between the PMA and TM regions (*p* < 0.001) and between the CT and TM regions (*p* = 0.006) (Figure 2), with the highest median value observed in the region with the lowest suicide RR (PMA) and the lowest median value observed in the region with the highest suicide RR (TM), which is in good agreement with the findings of most ecological studies, as already highlighted. Thus, the actual impact of prolonged continuous Li exposure on suicide RR remains an open question. Could the continuous chronic exposure of the population to Li through drinking water, even with such low concentrations (only a few µg/L) and small differences, be reflected in different suicide RRs? This is in contradiction with the findings of Knudsen et al. [19], who reported no “protective” effect for Li concentrations in drinking water below 30 µg/L. A recent study in Switzerland reporting low Li concentrations in drinking water (median = 2.86 µg/L; range = 0.06–37.1 µg/L) also found no association between Li levels and suicide rates [21].

A positive correlation was observed between the Li concentration in urine and drinking water, but it was very weak (ρ = 0.174; *p* = 0.002). This suggests that other sources may be more relevant for total daily Li intake, especially when drinking water contains such low concentrations of Li, as observed in the present study. When considering only the participants who provided drinking water samples with Li concentrations higher than 10 µg/L, the correlation became stronger (ρ = 0.451; *p* = 0.046; n = 20). Major dietary sources of Li include cereals, potatoes, tomatoes, cabbage, and certain mineral waters [25]. A vegetarian diet rich in grains and vegetables typically provides more Li than a diet high in animal products, but this can vary significantly by geographical location due to the uneven distribution of Li in the Earth’s crust and, consequently, its concentration in plants [26]. Grains and vegetables can account for 66–90% of the daily Li intake [25].

The participants were also asked to provide a sample of environmental water (water from rivers, streams, lakes, wells, mines, natural springs, etc.) collected in their region of residence. In nature, Li is found as part of various minerals and is unevenly distributed [3,5]. From rocks, Li is released into water and soil, from where it is absorbed by plants, thus entering the food chain [5]. The analysis of environmental waters can, therefore, provide valuable information on the degree of potential exposure to Li by the local population. A total of 298 environmental water samples were analysed. The median (P25–P75) Li concentrations were 2.81 (1.83–6.10), 4.48 (1.82–8.61), and 2.77 (1.35–5.64) µg/L for the PMA, CT, and TM regions, respectively (Figure 2), with no statistically significant differences between them (*p* = 0.140). No correlation was found between Li concentration in the environmental waters and urine (ρ = −0.036; *p* = 0.694).

In any case, it is important to highlight that, somewhat contrary to what might be expected, the concentration of Li in the environmental waters was of the same order of magnitude as that of the Li in the drinking water (just a few µg/L) (Figure 2).

Taken together, these results suggest that differences in suicide RR between the three regions studied are unlikely due to differences in natural Li exposure. These findings contradict the results of several ecological studies [11,12,13,14,15] that led some authors to consider the supplementation of drinking water with Li as a public policy for suicide prevention [27,28]. However, more robust and comprehensive studies evaluating the relationship between natural Li exposure and suicide risk are needed to better define its potential protective role before considering universal drinking water supplementation. The potential negative effects of excessive Li exposure should also be considered. Impaired renal concentrating ability is the most common side effect of chronic Li therapy, but animal studies have shown additional negative effects of chronic Li exposure on other organs [29]. In 2008, the U.S. Environmental Protection Agency (EPA) derived a chronic provisional oral reference dose (p-RfD) for Li of 2 µg/kg/day (140 µg/day for a 70 kg individual) using the lowest-observed-adverse-effect level of 0.6 mmol/L of Li in serum and an uncertainty factor of 1000 (a factor of 10 because no no-observed-adverse-effect level (NOAEL) was available in the literature; a factor of 10 to protect susceptible individuals; and a factor of 10 to account for database insufficiencies) [29]. For comparison, daily Li intake was estimated at 48.2 µg/day in France [30], 18.2 µg/day in Italy [9], and 17 µg/day in the UK [31], while in the population of the Canary Islands, it was estimated at 3674 µg/day [32]. Finally, postmortem measurement of Li levels in the brain tissue of individuals who committed suicide (and in controls) would be an important contribution to the study of this issue since the problem may lie in the access of Li to brain cells.

The present study has some methodological limitations. The convenience sampling method used to recruit participants does not ensure a representative sample of the populations under study. Additionally, other factors such as occupation, marital status, education, and income levels also appear to influence suicide risk and suicidal behaviours [33,34,35]. Furthermore, there were significant statistical differences in the participants’ gender and age distribution between regions, which may hinder comparisons. On the other hand, urinary Li levels are expected to vary considerably depending on diet, so analysing a larger number of samples collected at different times of the year and obtaining multiple samples from the same participant would be beneficial. Finally, the urinary Li levels were not normalized for urinary creatinine excretion.

## 5. Conclusions

In this study, Li levels in drinking water, environmental water, and urine of individuals living in three Portuguese regions with different relative risks (RRs) of suicide were compared.

A statistically significant difference was observed in the Li levels in the drinking water between the region with the lowest RR (Porto Metropolitan Area), which presented the highest median value, and the region with the highest RR (Trás-os-Montes, Northeast Portugal), which presented a lower median value (3.10 vs. 1.24 µg/L, respectively). Regarding the Li levels in the environmental waters—measured to characterize the overall local environment in terms of Li levels and, ultimately, the supply of Li through the consumption of locally produced foods—no statistically significant differences were observed. Overall, both the drinking water and environmental waters had Li concentrations well below the levels that have been considered “protective” in ecological studies.

The main novelty of this study is the measurement of urinary Li concentrations in residents from the three regions, assuming that this allows the reliable assessment of total Li exposure, regardless of its source (drinking water, food, etc.). To our knowledge, this is the first study to compare the urinary Li concentration among residents of regions with different suicide RRs. Urinary Li concentrations in the three regions (PMA, low RR; CT, intermediate RR; TM, high RR) showed no statistically significant differences after applying the Bonferroni correction for multiple testing. Additionally, given the overall low Li levels in the drinking water—which does not appear to be a major dietary source of Li—the correlation between urinary Li concentration and drinking water Li concentration was weak.

## Figures and Tables

**Table 1:** Study participant characterization.

	Regions			
	PMA (Low RR)	CT (Intermediate RR)	TM (High RR)	*p*-Value
Total, n (%)	91 (29.3%)	130 (41.8%)	90 (28.9%)	-
Sex Women, n (%)	65 (71.4%)	78 (60.0%)	65 (72.2%)	0.019 ^#^
Men, n (%)	22 (24.2%)	46 (35.4%)	16 (17.8%)	
No response, n (%)	4 (4.4%)	6 (4.6%)	9 (10.0%)	
Age, mean (SD)	43 (17)	49 (17)	46 (14)	0.016 *

^#^ Differences in the sex distribution across regions (Chi-square test of independence); * one-way ANOVA test.

**Table 2:** Sources of drinking water and sources of environmental water.

	Regions				
	PMA (Low RR)	CT (Intermediate RR)	TM (High RR)	All Regions	*p*-Value
**Drinking water source:**					<0.001 *
Public supply network	76 (85%)	85 (68%)	83 (97%)	244 (81%)	
Well, borehole, other groundwater source	12 (14%)	4 (3%)	2 (2%)	18 (6%)	
Other (non-specified source)	1 (1%)	36 (29%)	1 (1%)	38 (13%)	
**Environmental water source:**					<0.001 *
River, lake, other surface water source	1 (1%)	19 (15%)	9 (12%)	29 (10%)	
Public fountain	24 (32%)	74 (57%)	37 (51%)	135 (49%)	
Well, borehole, other groundwater source	32 (45%)	14 (11%)	21 (29%)	67 (24%)	
Other (non-specified source)	18 (24%)	23 (18%)	6 (8%)	47 (17%)	

* Differences in the sources of water across the three regions (Chi-square test of independence).

## Data Availability

The original contributions presented in this study are included in this article/the Supplementary Material. Further inquiries can be directed to the corresponding author.

## Supplementary Materials

The following supporting information can be downloaded at https://www.mdpi.com/article/10.3390/nu17071283/s1: Figure S1. Spatiotemporal clusters of high (left) and low (right) suicide death rates across Portugal (continental); Table S1. Analytical quality control results.
