# Supplementary material for: Association Between Natural Lithium Exposure and Suicide Rate: An Ecological and Biomonitoring Study in Portugal
Source: Nutrients. 2025 Apr 7;17(7):1283. doi: 10.3390/nu17071283 (PMC11990145; doi:10.3390/nu17071283)
Supplement: Supplementary file 1 [file nutrients-17-01283-s001.zip › nutrients-3496729-SI.pdf]

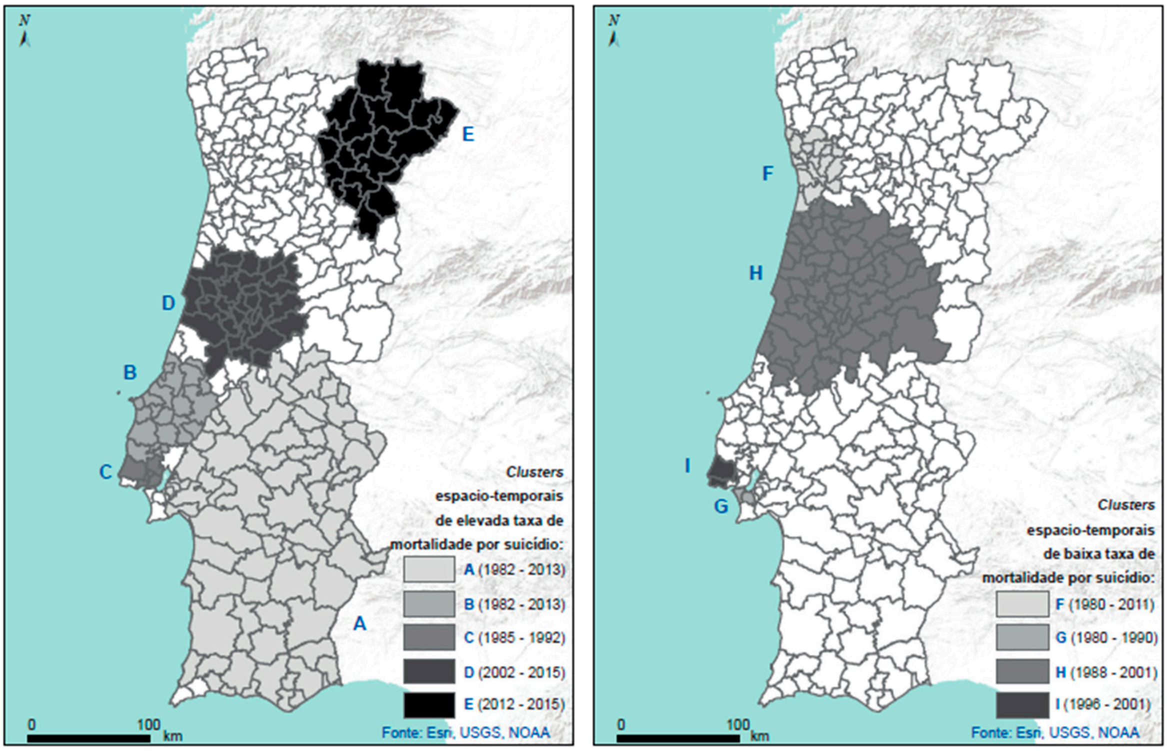

Figure S1. Spatiotemporal clusters of high (left) and low (right) suicide death rates across Portugal (continental); source: Mortalidade por suicídio nos municípios de Portugal Continental: Evolução espácio-temporal entre 1980 e 2015 [article in portuguese]. Acta Med Port 2018, 31, 38-44, doi: 10.20344/amp.9423.

Table S1. Analytical quality control results.

QC sample / Certified reference material	LOD (µg/L)	Result (µg/L) mean (SD)	Certified value (µg/L)	95% CI (µg/L)	Recovery	Within-run precision	Between-run precision
Seronorm™ Urine L-1	0.515	4.82 (0.16)	5.2*	-	93%	2.4%	3.3%
Seronorm™ Urine L-2		4.92 (0.16)	5.2*	-	95%	1.9%	3.2%
EnviroMAT Drinking Water (1:20 dilution)#	0.272	407 (8)	403	392 – 415	101%	1.5%	1.9%
EnviroMAT Drinking Water (1:100 dilution)#		403 (17)			100%	3.4%	4.2%

* "Indicative values"; 95% confidence interval (CI) is not provided by the Quality Control (QC) samples supplier. #Diluted 1:100 as recommended by the manufacturer and then diluted 1:20 or 1:100 before analysis by ICP-MS. LOD = limit of detection.
